# Navigating the Impossible Gallbladder: Bailout Strategies and Culture of Safety in Laparoscopic Cholecystectomy

**DOI:** 10.7759/cureus.104915

**Published:** 2026-03-09

**Authors:** Fatima Rauf, Sophia Echevarria, Suman Aamir, Iffat Noureen, Muhammad Rawal Saeed, Muhammad Hanif, Huma Sabir Khan, Usman Qureshi

**Affiliations:** 1 Surgical Unit II, Benazir Bhutto Hospital, Rawalpindi Medical University, Rawalpindi, PAK; 2 General Practice, Universidad Mayor de San Simon, Cochabamba, BOL; 3 Department of Surgery, Regional Headquarter Hospital, Skardu, Skardu, PAK

**Keywords:** bailout strategies, b-safe landmark, culture of safety, difficult laparoscopic cholecystectomy, gallstone disease, hepatobiliary system, indocyanine green fluorescence, laparoscopic subtotal cholecystectomy, pathogenesis of gall stones, sulcus of rouvière

## Abstract

Cholecystectomy is one of the most frequently performed abdominal procedures worldwide, yet it continues to carry significant risks, particularly bile duct injury and hemorrhage, which can have devastating consequences. The complexity of the operation arises largely from the variable anatomy of the hepatobiliary system, the presence of acute or chronic inflammation, and the surgeon’s ability to recognize when anatomy is unsafe for dissection. A comprehensive understanding of gallstone pathogenesis, relevant surgical anatomy, and potential complications forms the foundation for safe practice. Over recent decades, the evolution of a “culture of safety” in cholecystectomy has emphasized universal principles such as the critical view of safety, B-SAFE landmarks (Bile duct, Sulcus of Rouvière, Arterial pulsations, Fissure, Enteric structure), and the role of Rouviere’s sulcus in orientation. These strategies are complemented by the use of stopping rules, second opinions, and intraoperative imaging modalities such as cholangiography, laparoscopic ultrasound, and indocyanine-green fluorescence to reduce misidentification. Despite these precautions, situations frequently arise in which the critical view of safety cannot be achieved. In such cases, surgeons must be prepared to employ bailout strategies, including abortion of the procedure, conversion to open surgery, tube cholecystostomy, subtotal cholecystectomy, and the fundus-first approach. Subtotal cholecystectomy in particular has emerged as the most definitive bailout, supported by strong evidence and international guidelines, while other approaches provide situational pathways to safety. This review synthesizes current knowledge across pathogenesis, anatomy, complications, safe cholecystectomy principles, intraoperative adjuncts, and bailout procedures. By integrating technical evidence with consensus recommendations, it highlights the critical importance of surgical judgment and adherence to safety protocols in minimizing complications and ensuring favorable patient outcomes.

## Introduction and background

Cholecystectomy is among the most commonly performed surgical procedures worldwide and is considered the definitive treatment for symptomatic gallstone disease. The introduction of laparoscopic cholecystectomy more than three decades ago revolutionized biliary surgery, offering faster recovery and improved patient outcomes compared to the open approach [1]. However, it also brought new challenges, most notably the risk of bile duct injury, which can have devastating consequences for patients and significant medicolegal implications for surgeons [2,3].

The frequency and severity of such complications underscore the importance of adopting strategies that prioritize safety over procedural completion. Central to this is the development of a “culture of safety” in cholecystectomy, which emphasizes meticulous anatomical identification, recognition of risk factors, and readiness to employ alternative strategies when safe dissection is not possible [4]. The Critical View of Safety (CVS) has become the cornerstone of safe practice, while adjunctive measures such as intraoperative imaging and bailout techniques further enhance safety in difficult cases [5,6].

Despite advances, difficult gallbladders continue to challenge even experienced surgeons, necessitating a systematic approach that combines anatomical knowledge, technical judgment, and evidence-based strategies. This review synthesizes the pathogenesis of gallstones, surgical anatomy, complications, safe operative principles, and bailout procedures. Unlike previous reviews that address these aspects separately, this article integrates these elements within a practical framework focused on intraoperative decision-making in difficult cholecystectomy. Particular emphasis is placed on bailout strategies and their appropriate indications when the critical view of safety cannot be achieved. By consolidating current evidence, it aims to highlight practical strategies that reduce operative risk and support safer outcomes in cholecystectomy. 

## Review

Pathogenesis of cholelithiasis

Gallstone disease is one of the most common gastrointestinal disorders worldwide and represents the foundation upon which the need for cholecystectomy arises. The formation of gallstones is a complex, multifactorial process influenced by metabolic, genetic, infectious, and environmental factors [7]. Cholesterol supersaturation of bile remains the cornerstone of gallstone pathogenesis, although pigment stones and mixed stones also occur.

**Figure 1:**
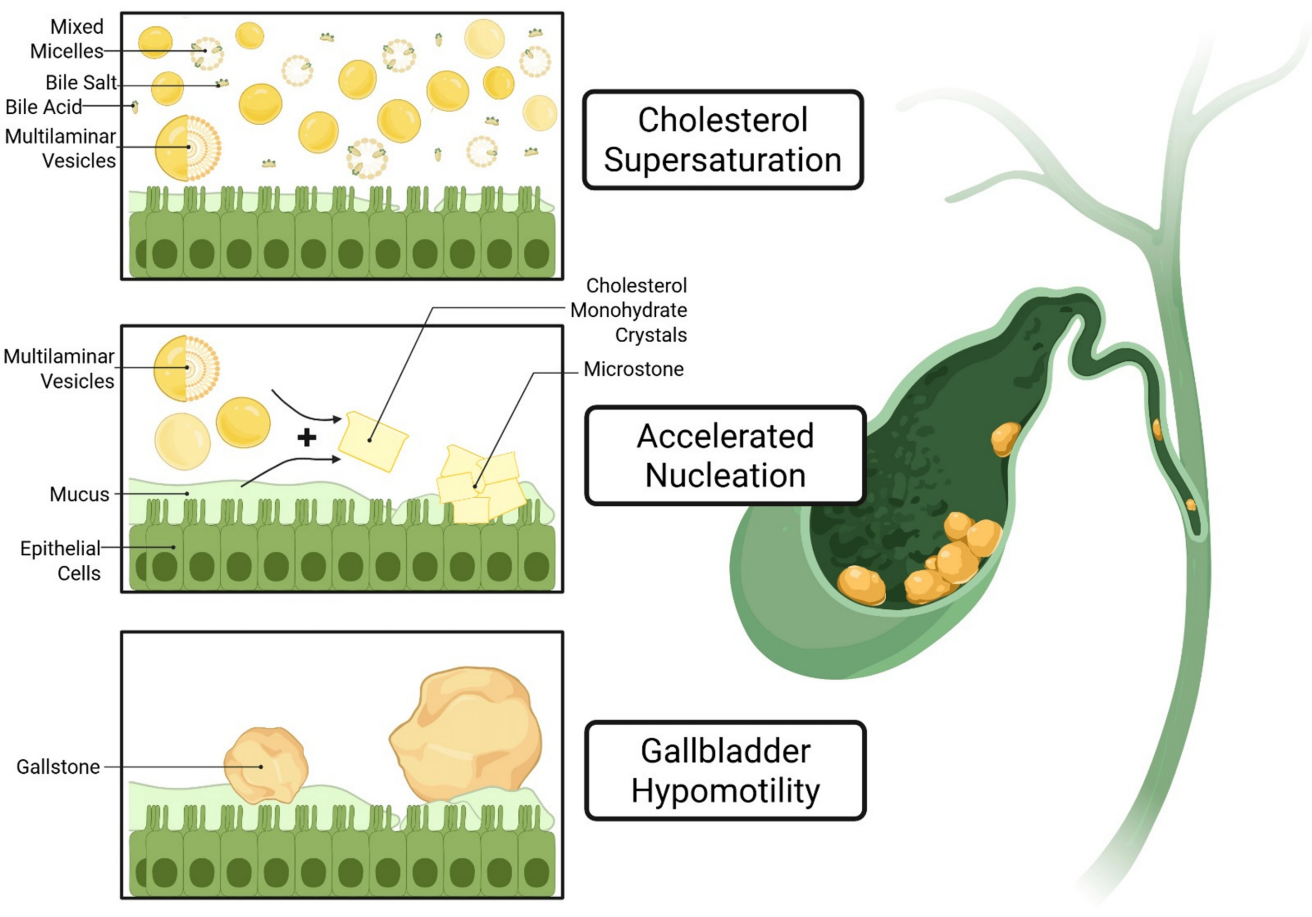
Pathogenesis of cholelithiasis Image Credit: Sophia Echevarria (author); created in BioRender (https://biorender.com/72z6pbd)

Cholesterol gallstones form when bile becomes supersaturated with cholesterol relative to bile salts and phospholipids. Under normal physiology, cholesterol is solubilized in bile micelles composed of bile salts and lecithin. When supersaturation occurs, cholesterol precipitates and nucleates into crystals [8]. This process is facilitated by hypomotility of the gallbladder, impaired emptying, and stasis, which allow cholesterol crystals to aggregate and grow. These processes interact in a self-propagating cycle. Supersaturation induces mucin hypersecretion and dysmotility, which further impair bile salt cycling, contract the bile salt pool, and increase bile hydrophobicity (Figure 1). The resulting changes accelerate nucleation, destabilize vesicles, and worsen gallbladder dysfunction, driving lithogenesis [9].

Pigment stones arise from increased bilirubin turnover or impaired bilirubin metabolism. Black pigment stones occur in sterile bile and are associated with chronic hemolytic states, such as hereditary spherocytosis, thalassemia, and sickle cell anemia [10]. Brown pigment stones typically develop in the presence of infection or stasis, commonly seen in the biliary tree of East Asian populations. These stones are composed of calcium bilirubinate and are strongly associated with parasitic or bacterial colonization, which provides β-glucuronidase activity, catalyzing unconjugated bilirubin precipitation [11].

Risk factors for gallstone formation include female sex, increasing age, obesity, metabolic syndrome, rapid weight loss, pregnancy, multiparity, and use of estrogen or clofibrate [12]. Genetic predisposition plays a strong role, with variants in genes regulating cholesterol transport and hepatic bile secretion implicated in stone formation [7]. Family clustering studies and twin studies further support this heritability.

Epidemiologically, cholesterol stones dominate in Western countries, accounting for nearly 80-90% of cases, while pigment stones are more prevalent in Asian and African populations [8]. Dietary factors, particularly high caloric intake, refined carbohydrates, and low fiber consumption, also contribute [13].

The pathophysiology of gallstone disease underscores why gallbladder removal is often required for symptomatic patients. Understanding the metabolic and biochemical origins of stone formation helps surgeons appreciate that cholecystectomy addresses the sequelae of gallstones rather than their root metabolic causes.

Gallbladder anatomy

A thorough understanding of gallbladder anatomy remains the cornerstone of safe laparoscopic cholecystectomy, as even subtle anatomical differences can give rise to intraoperative confusion, hemorrhage, or catastrophic bile duct injury (BDI). The gallbladder is a pear-shaped organ nestled in a fossa between hepatic segments IVB and V, measuring approximately 7-10 cm in length and 3-4 cm in diameter. It is composed of four distinct anatomical parts: the fundus, the body, the infundibulum or neck, which can form Hartmann’s pouch when distended with stones; and finally, the cystic duct, which joins the common hepatic duct (CHD) to form the common bile duct (CBD) [14].

Arterial supply is primarily derived from the cystic artery, usually a branch of the right hepatic artery (RHA) coursing through Calot’s triangle; however, up to 25% of gallbladders exhibit atypical arterial supply, originating from the left hepatic, gastroduodenal, or superior mesenteric arteries. Venous drainage occurs via cystic veins into the portal vein or directly into hepatic sinusoids. Lymphatic drainage commences at the neck through cystic (Lund's) nodes and progresses toward hepatic and celiac lymph nodes [14,15].

Hepatocystic Triangle

The hepatocystic triangle lies on the undersurface of the liver, bordered by the CHD medially, the cystic duct caudally, and the liver superiorly. It contains the cystic artery, variable RHA branches, the cystic lymph node, lymphatics, and connective tissue. Unlike Calot’s triangle, the cephalad boundary is the liver surface, not the cystic artery [16]. This area is the main target for dissection during laparoscopic cholecystectomy, with the cystic lymph node serving as a key landmark, though inflammation or scarring may obscure anatomy in difficult cases [17].

Cystic Plate

The cystic plate is a flat ovoid fibrous sheet that forms part of the liver’s plate system, lying in the gallbladder bed and continuous with the liver capsule of segments 4 and 5 [15]. Toward the hepatic hilum, it narrows into a cord-like structure that joins the sheath of the right portal pedicle. It is normally concealed by the gallbladder and becomes visible as a whitish-grey structure once the gallbladder is dissected off. Exposure of the lower part of the cystic plate is crucial for achieving the CVS, since anomalous ducts or arteries may pass here and risk being injured if not recognized. Surgeons must stay close to the gallbladder wall during dissection, leaving areolar tissue on the cystic plate, as breaching it can lead to bleeding from liver parenchyma or bile leaks from sub-vesical ducts [18,19]. This risk is higher in chronic cholecystitis, where adhesions obscure dissection planes. In contracted gallbladders, the cystic plate length shortens, and careless dissection may enter the right portal pedicle, especially with the fundus-first approach, leading to serious vascular or biliary injury [19].

Rouviere’s Sulcus

Rouviere’s sulcus is a 2-5 cm groove on the underside of the right liver lobe, usually visible in about 80% of patients and often containing the right portal pedicle or its branches. It becomes most apparent during laparoscopic cholecystectomy when the gallbladder neck is retracted towards the umbilical fissure [20-22]. Clinically, it serves as a reliable landmark for identifying the CBD plane, guiding safe dissection in the hepatocystic triangle. All dissections should remain ventral and cephalad to the sulcus to avoid bile duct injury [20].

Umbilical Fissure

The umbilical fissure lies between the left lateral (segments 2, 3) and left medial (segment 4) sections of the liver, housing the falciform ligament and ligamentum teres. It serves as a constant anatomical landmark, assisting surgeons in orientation during difficult laparoscopic cholecystectomy [23]. Segment 4, located between the umbilical fissure and the gallbladder, also provides a key reference point, with dissection in the hepatocystic triangle performed cephalad to the R4U line. Proper assessment of its size and recognition of anomalies like hypoplasia or ectopic gallbladder is crucial to avoid bile duct injury [23,24].

Anatomic variations

Despite the seemingly straightforward anatomy, variations are not merely academic; rather, they are clinical minefields that directly affect surgical safety. The hepatocystic triangle is the main operative field in laparoscopic cholecystectomy, and knowledge of its anatomic variations is essential to avoid BDI and vascular injuries.

The cystic artery, usually single and arising from the RHA, typically traverses the hepatocystic triangle and divides into superficial and deep branches. However, variations include an anterior course to the bile duct (17.9%), a short cystic artery (<1 cm, 9.5%), multiple arteries (8.9%), or a low-lying artery passing below the cystic duct (4.9%). A short cystic artery, if unrecognized, may lead to inadvertent RHA injury, which can be avoided by keeping the dissection close to the gallbladder on the right of the cystic lymph node [16,17]. In rare cases, the cystic artery may arise from the gastroduodenal or left hepatic artery, lying outside the hepatocystic triangle. The RHA itself usually passes posterior to the CHD (87%), but replaced or accessory RHAs are not uncommon. A replaced RHA may mimic a large cystic artery as it courses close to the cystic duct and gallbladder, predisposing it to injury. In some cases, the RHA may follow a tortuous course, known as Moynihan’s hump or Caterpillar turn, bringing it dangerously close to the gallbladder and cystic duct, thereby increasing the risk of vascular injury during dissection [17].

Ductal anomalies relevant to laparoscopic cholecystectomy mainly involve the cystic duct and the right hepatic ductal system [25]. The cystic duct is usually 2-4 cm long and 2-3 mm wide, but it can be very short, congenitally absent (rare), or unusually long (>5 cm). Normally, it joins the CHD at an angle (75%), but may follow a parallel (20%) or spiral (5%) course [16,25]. Variations include entry into the right hepatic duct (0.6-2.3%), anomalous right sectional ducts, or even low insertion near the ampulla. Rarely, duplication of the CBD occurs [26]. Clinically, not all of the cystic duct needs to be dissected, as excessive pursuit increases the risk of bile duct injury, especially with parallel insertions. A non-visualized cystic duct may indicate it is short, effaced by stones, or obscured by Mirizzi syndrome, requiring caution and possibly bail-out strategies. If two ducts are seen, they should not be assumed to be double cystic ducts without clarification, ideally with intraoperative cholangiography (IOC), since one could represent the CBD. A dilated cystic duct (up to 5 mm) due to stones may also mimic the CBD, and difficulty in occluding it with clips should raise suspicion [27]. Similarly, anomalous low insertion of a right sectional duct, especially the posterior one, can be at risk of injury if unrecognized, highlighting the importance of obtaining the CVS or intraoperative imaging before division [25].

From a clinical perspective, these anatomic variations not only increase operative complexity but also alter the risk-benefit balance of laparoscopic cholecystectomy. In cases with severe inflammation or dense adhesions obscuring normal planes, unrecognized variations amplify the risk of inadvertent ductal or vascular injury. This reinforces the importance of Strasberg’s CVS, which mandates the unequivocal identification of exactly two and only two structures-the cystic duct and the cystic artery-before transection. When CVS cannot be achieved due to distortion by inflammation or variant anatomy, surgeons must rely on bailout strategies [28-30].

Complications of cholecystectomy

Although laparoscopic cholecystectomy has become one of the most frequently performed operations in general surgery, it is not without significant risks. Complications can be immediate or delayed, minor or catastrophic, and their prevention requires anticipation, knowledge of risk factors, and meticulous surgical technique [24].

Injuries to the Biliary System

Biliary injuries remain the most feared complication, occurring in about 0.4-1.5% of cases, with a higher incidence in laparoscopic procedures compared to open surgery [31-33]. The major cause is misidentification of biliary anatomy, often due to inflammatory adhesions, distorted anatomy, or surgeon inexperience. Variations such as an aberrant RHD or accessory ducts (ducts of Luschka) increase the risk. Mechanisms include incorrect clipping, slipped ligatures, and thermal injury from diathermy [34].

Clinical consequences include bile leaks, strictures, and transections. Bile leaks may originate from the cystic duct stump, ducts of Luschka, or partially injured hepatic ducts. Large injuries involving the CHD or CBD can cause severe morbidity. Patients may present with persistent bilious drain output, abdominal pain, jaundice, fever, or sepsis [35].

Long-term sequelae include chronic strictures, recurrent cholangitis, secondary biliary cirrhosis, and portal hypertension [36]. Classification systems guide assessment and management [37,38]. Bismuth classification IS based on the level of injury/stricture in relation to the hepatic duct confluence (Table 1). Strasberg classification (Types A-E) is specific for laparoscopic injuries (Table 2). 

**Table 1:** Bismuth classification of iatrogenic biliary injuries during laparoscopic cholecystectomy CHD: common hepatic duct; RHD: right hepatic duct; CBD: common bile duct. Table Source: Abdelgawad et al., 2023 [34]; Published under CC BY 4.0 Attribution 4.0 International Deed (https://creativecommons.org/licenses/by/4.0/)

Types	Description
Type 1	Low CHD injury, with a length of the CHD stump of > 2 cm
Type 2	Middle injury, length of CHD < 2 cm
Type 3	Hilar injury, no remaining CHD, but the confluence is preserved
Type 4	Hilar injury, with involvement of confluence and loss of communication between right and left hepatic ducts
Type 5	Aberrant RHD injury with or without concomitant CHD injury

**Table 2:** Strasberg classification of iatrogenic biliary injuries during laparoscopic cholecystectomy Table Source: Eum et al., 2014 [39]; Published under CC BY-NC 4.0 Attribution-NonCommercial 4.0 International Deed (https://creativecommons.org/licenses/by-nc/4.0/)

Class	Description
A	Injury to small ducts in continuity with biliary system, with cystic duct leak
B	Injury to sectoral duct with consequent obstruction
C	Injury to sectoral duct with consequent bile leak from a duct not in continuity with biliary system
D	Injury lateral to extrahepatic ducts
E1	Stricture located > 2 cm from bile duct confluence
E2	Stricture located < 2 cm from bile duct confluence
E3	Stricture located at bile duct confluence
E4	Stricture involving right and left bile ducts
E5	Complete occlusion of all bile ducts

Prompt recognition is critical. Early management may involve endoscopic retrograde cholangio pancreatography (ERCP) with stenting for minor leaks, but major transections often require hepaticojejunostomy or complex biliary reconstruction [40]. These injuries represent one of the most litigated complications in surgery and are associated with lifelong morbidity.

Vascular Complications

Vascular injuries, though less common than biliary injuries, carry a high risk and are often associated with vasculobiliary injury [41]. The RHA is most frequently affected due to its close proximity to the cystic duct and artery. Injuries may include ligation, thrombosis, or pseudoaneurysm formation. RHA thrombosis can lead to ischemia of the bile ducts, biloma formation, hepatic necrosis, or segmental atrophy. Portal vein injury, though rare, has grave consequences, including liver infarction and massive hemorrhage. Cystic artery stump bleeding may occur from slipped clips, presenting with intraperitoneal hemorrhage or hemobilia. Other sources include bleeding from liver bed veins, abdominal wall vessels (e.g., epigastric arteries during trocar insertion), or rarely major vessels such as the aorta or inferior van cava (IVC) [42]. Clinically, vascular injuries may manifest as intraoperative hemorrhage, postoperative shock, dropping hemoglobin, or ischemic changes in the liver. Management ranges from angiographic embolization for pseudoaneurysm/hemorrhage to surgical repair or hepatic resection in severe cases.

Gallstone-Related Complications

Gallstone-related problems include retained or recurrent stones in the biliary tract, leading to jaundice, cholangitis, or pancreatitis. Dropped stones during surgery occur in up to 30-36% of cases, usually remaining asymptomatic [43]. In rare cases, they cause intra-abdominal abscesses, sinus tracts, or fistulae years later [44].

Postoperative Collections

Hematomas, bilomas, and abscesses are recognized postoperative sequelae. Hematomas usually result from inadequate hemostasis or slipped ligatures. Bilomas are localized bile collections from leaks [45]. Abscesses may occur from infected collections or dropped stones. Ultrasound and CT are useful for detection, and management may involve percutaneous drainage or reoperation in severe cases [46].

Miscellaneous Complications

Other complications include wound infections, port-site hernias, inadvertent bowel injuries (thermal or trocar-related), and, rarely, clip migration into the biliary tree, causing obstruction or cholangitis [47-49].

Morbidity and mortality connected with laparoscopic cholecystectomy are low, generally 11.1% and 0.2%, respectively, but this increases significantly in elderly patients, those undergoing emergency procedures, and in the presence of severe comorbidities [50].

From a medicolegal standpoint, bile duct injury is the leading cause of litigation in general surgery [51]. Preventive strategies include adherence to the CVS, liberal use of intraoperative imaging, recognition of stopping rules, and appropriate bailout procedures when safe progression cannot be achieved [4].

Safe cholecystectomy

Safe cholecystectomy requires a systematic approach that emphasizes anatomical awareness, meticulous technique, and adherence to principles designed to minimize the risk of bile duct and vascular injury. The concept of safety in laparoscopic cholecystectomy has evolved into a global framework supported by surgical societies such as the Society of American Gastrointestinal and Endoscopic Surgeons (SAGES), the World Society of Emergency Surgery (WSES), and the Tokyo Guidelines, often summarized under the “Culture of Safety in Cholecystectomy” (COSIC) [43]. The principle is simple: the aim of surgery is not merely gallbladder removal, but its safe removal, even if that requires modification of the procedure or abandonment.

Safe Dissection

The first element of safe dissection is exposure. Proper positioning of the patient, adequate pneumoperitoneum, and optimal port placement are fundamental. The gallbladder should be retracted cephalad and laterally to open up the hepatocystic triangle. Retraction must be balanced to avoid tenting of the bile duct, which can lead to misidentification and injury [52]. Before entering Calot’s triangle, surgeons should ensure clear visualization of landmarks such as the falciform ligament, segment IV of the liver, Rouviere’s sulcus, and the gallbladder infundibulum. These structures orient dissection and help maintain awareness of the biliary confluence.

Safe Zones and Anatomical Landmarks

Several extrabiliary landmarks have been emphasized to guide safe surgery. The B-SAFE mnemonic, standing for bile duct, sulcus of Rouviere, artery (RHA), fissure (umbilical fissure), and enteric structures, helps surgeons orient themselves even in difficult conditions. Among these, Rouviere’s sulcus serves as a critical horizontal line, with dissection advised to remain ventral to this plane to avoid injury to the CBD [53]. Similarly, the “R4U line” connects the roof of segment IV (R4) to the base of the umbilical fissure (U), and dissection above this line remains within a safer zone [54]. The cystic plate, a fibrous plane forming the gallbladder bed, also serves as a natural limit to dissection during both antegrade and fundus-first techniques.

Critical View of Safety

The CVS, described by Strasberg, remains the gold standard for safe identification of the cystic duct and artery [55]. To achieve CVS, three criteria must be fulfilled: clearance of fat and fibrous tissue from the hepatocystic triangle, separation of the lower third of the gallbladder from the liver bed to expose the cystic plate, and confirmation that only two structures enter the gallbladder. Division of any structure without this confirmation is unsafe [56]. Importantly, CVS is a technique of exposure, not of dissection, and it must be documented either by still images or intraoperative video [57]. Photo documentation not only reinforces surgical education but also serves as medicolegal protection in the event of complications [58].

The Time-Out Principle

Another protective measure is the deliberate intraoperative pause or “time-out.” This allows the surgical team to reassess the dissection and decide whether CVS can realistically be achieved. Common pause points include before entering the hepatocystic triangle, when anatomy appears unclear, or immediately before dividing ductal or vascular structures [30,59]. These pauses encourage reflection and team communication, which can prevent hasty or unsafe decisions. By formalizing a moment of reassessment, time-outs reinforce the principles of CVS and Strasberg’s framework, giving the surgeon an opportunity to avoid injury through timely course correction.

Energy Use in Dissection

Judicious use of energy devices is another principle of safety. Thermal spread from monopolar cautery or ultrasonic devices can injure adjacent ducts or bowel, sometimes with delayed presentation [60]. Energy use should be kept close to the gallbladder wall and limited around Calot’s triangle. Where necessary, blunt and cold dissection techniques may provide greater safety.

Safety Measures Endorsed by the Strasberg’s Framework and Society

Building on the CVS, Strasberg proposed in 2019 a broader framework for safe laparoscopic cholecystectomy, consisting of three stages [61]. First, surgeons should strive for clear anatomical identification, ideally by achieving CVS. Second, they must recognize the “inflection point,” which is the point where safe identification is no longer possible due to distorted anatomy or dense inflammation. Third, once this inflection point is reached, surgeons should not persist but instead adopt alternative strategies.

Strasberg’s three-step framework emphasizes judgment as much as technical ability. Safe laparoscopic cholecystectomy is not defined only by meticulous dissection but also by the ability to recognize limits and adapt. By integrating CVS into this stepwise decision-making process, Strasberg provided surgeons with a practical roadmap that balances technical precision with situational awareness.

International Guidelines

International guidelines repeatedly stress that safe cholecystectomy is grounded in situational awareness, not rote adherence to technique. The SAGES Safe Cholecystectomy Guideline, WSES position statements, and Tokyo Guidelines 2018 all emphasize a universal culture of safety built upon CVS, liberal use of imaging, clear exposure, awareness of extrabiliary landmarks, and recognition of stopping rules. Importantly, these guidelines acknowledge that surgeon experience and judgment remain decisive in applying these principles to real-life scenarios [4,62,63]. The cumulative effect of these strategies is a layered defense against bile duct injury. Ultimately, safe cholecystectomy is not a single maneuver but a philosophy of care: patient safety above completion of the planned operation, every time.

Techniques and strategies for safe completion of difficult cholecystectomy

Techniques and strategies for the safe completion of difficult cholecystectomies have evolved through decades of collective experience and analysis of adverse outcomes. Laparoscopic cholecystectomy, though now the standard of care, carries a higher risk of bile duct injury compared with the open era. Preventing such injury depends not only on technical skill but also on judgment, awareness, and structured decision-making frameworks that prioritize patient safety over the compulsion to complete the operation at all costs.

Recognizing Stopping Rules

A critical element in preventing major complications is the surgeon’s ability to recognize when the dissection has entered a “zone of danger.” Red flags include dense adhesions, severe acute inflammation, large impacted stones, Mirizzi syndrome, or chronic fibrosis. These conditions can obscure visualization, distort anatomy, and stall safe progress. Prudence requires the surgeon to pause (“time-out”), acknowledge the rising risk of bile duct or vascular injury, and reassess the operative plan before proceeding further [30]. Early recognition of these cues allows the surgeon to act at a point of safe return, before irreversible harm occurs.

Seeking a second opinion: Stopping rules, however, do not imply that the surgeon must always decide in isolation. Seeking a second opinion is a powerful but often underutilized safety measure. Misidentification of biliary anatomy, especially mistaking the CBD for the cystic duct, remains the leading cause of major bile duct injuries [60]. Evidence suggests that intraoperative consultation with a colleague can prevent such errors in nearly one-fifth of difficult cases [64,65]. A second surgeon can provide a fresh perspective, scrub in to assist, propose alternative strategies, or reinforce the decision to pursue an exit procedure. Importantly, this should not be viewed as a sign of weakness but as sound surgical judgment. Encouraging consultation across all levels of training reflects the philosophy of the COSIC, which emphasizes collaboration and collective responsibility [66].

Role of intraoperative imaging: Adjunctive intraoperative imaging represents another essential tool when anatomy is unclear. IOC is the most widely studied method, performed by cannulating the cystic duct and injecting contrast to delineate the biliary anatomy. While large database studies have not consistently shown that routine IOC reduces the overall rate of bile duct injury, multiple prospective studies and meta-analyses highlight its utility in acute cholecystitis, reoperative fields, and patients with abnormal liver function or ductal dilatation [67]. A meta-analysis including more than two million patients demonstrated that routine IOC was associated with a significantly lower rate of bile duct injury compared to selective use [68]. Beyond prevention, IOC enables early recognition of injuries and facilitates intraoperative management of choledocholithiasis. Its limitations include technical difficulty, added operative time, cost, and the requirement for fluoroscopic equipment [69]. For these reasons, current international guidelines recommend liberal but selective use, tailored to individual patient risk and anatomical uncertainty [4].

Laparoscopic ultrasound (LUS) offers a safe, radiation-free alternative, providing real-time imaging of the biliary tree. It avoids the logistical challenges of IOC and has particular value in patients where cannulation is difficult or contraindicated. Studies confirm its accuracy in identifying the cystic duct and CBD, though visualization of deep or intrahepatic ducts can be limited, and the technique is operator-dependent [70]. While not yet as widely adopted as IOC, LUS represents a valuable adjunct in difficult cases.

Near-infrared fluorescence cholangiography using indocyanine green (ICG) is an emerging modality that enhances intraoperative visualization of biliary structures. After intravenous administration, ICG is excreted in bile, allowing near-infrared imaging systems to highlight the cystic duct, CBD, and hepatic ducts in real time. Early studies suggest that ICG cholangiography reduces conversion rates, decreases the need for subtotal procedures, and lowers the risk of bile duct injury [71]. Limitations include shallow tissue penetration (5-10 mm), variable equipment availability, and the absence of large randomized trials. Despite these, it is increasingly being adopted in high-risk or complex cholecystectomies. The COSIC consensus acknowledges its promise as an adjunctive tool in cases of difficult anatomy [4,72].

Bailout strategies in difficult cholecystectomy

When, despite stopping rules, expert consultation, and adjunctive imaging, the critical view of safety cannot be achieved, the surgeon must adopt a bailout strategy. These approaches are not failures but deliberate, evidence-based alternatives designed to minimize harm. The principle is that safe completion of the operation is more important than anatomical radicality. The main bailout strategies include subtotal cholecystectomy (fenestrating or reconstituting), conversion to open surgery, fundus-first (top-down) approach, cholecystostomy tube drainage, and, in extreme cases, abandoning the procedure after drainage for damage control. Each of these strategies has unique technical considerations, advantages, limitations, and long-term outcomes, and their selection depends on intraoperative findings, surgeon experience, and patient comorbidities.

Abandoning the Procedure

In some rare instances, dissection during laparoscopic cholecystectomy becomes technically impossible, and persisting would cause more harm than good. In these circumstances, the safest choice is to abort the procedure altogether. Typically, this situation arises when only the gallbladder fundus is accessible while Calot’s triangle is completely obscured by adhesions, inflammation, scarring, or fistula formation. Hemodynamic instability during severe acute cholecystitis may also prompt abandonment. Intraoperative alternatives include laparoscopic cholecystostomy, which decompresses the gallbladder and controls sepsis, followed by delayed cholecystectomy after 6-12 weeks, or gallbladder aspiration with antibiotic instillation. A 19-year series involving 757 laparoscopic cholecystectomies reported aspiration in 5.3% of cases, with interval cholecystectomy successful in 72.5% and markedly lower conversion rates compared with standard procedures, without aspiration-related complications [73]. In extreme situations, the gallbladder may be left in situ and managed conservatively with antibiotics, with or without percutaneous drainage. Although such approaches require reoperation, they are justified when the alternative is catastrophic injury [63].

Conversion to Open Cholecystectomy

Conversion to open cholecystectomy remains a well-established option when laparoscopic progress cannot safely continue. Indications include uncontrolled hemorrhage, poor visualization of Calot’s triangle, intolerance to pneumoperitoneum, or suspected bile duct injury. Open access provides improved exposure, permits manual palpation, and facilitates hemostasis. However, conversion itself is not a bailout. It is only a change of access. The definitive bailout lies in what follows, such as open subtotal cholecystectomy, tube drainage, or conventional open cholecystectomy. Outcomes of conversion are not uniformly better. Indeed, converted cases are associated with a higher incidence of bile duct injury, with some series reporting up to 100-fold increased risk compared with straightforward laparoscopic cholecystectomy [74,75]. Compounding this is the decline in open biliary surgery exposure among younger surgeons, making these technically demanding operations increasingly challenging [74]. Retrospective studies confirm bile duct injury rates exceeding 3% in converted cases, compared with negligible rates in laparoscopic subtotal cholecystectomy. In addition to safety concerns, conversion adds a financial burden. Large database analyses report 25-30% higher hospital charges compared with prolonged but completed laparoscopic operations [76]. Thus, conversion must be recognized as a bridge, not an endpoint, and applied judiciously with senior assistance when necessary.

Tube Cholecystostomy

Tube cholecystostomy is another bailout technique when dissection is unsafe, especially in critically ill or unstable patients. Intraoperative tube drainage of the gallbladder achieves decompression, reduces intraluminal pressure, and controls sepsis, thereby stabilizing the patient until interval cholecystectomy can be attempted. Unlike percutaneous cholecystostomy performed radiologically in non-operative candidates, tube cholecystostomy is a deliberate intraoperative bailout. The CHOCOLATE (CHolecystectomy versus Percutaneous catheter draiNage for Acute Cholecystitis in High-Risk pATiEnts) randomized trial showed that laparoscopic cholecystectomy was superior to percutaneous drainage in high-risk acute cholecystitis, with fewer complications and lower recurrence of biliary events [77]. Tokyo Guidelines 2018 endorse cholecystostomy for critically ill patients unfit for immediate surgery, while reaffirming that early laparoscopic cholecystectomy remains the standard when feasible [62]. Despite its temporizing nature, intraoperative tube cholecystostomy has an important role as a lifesaving option.

Subtotal Cholecystectomy

Among bailout techniques, subtotal cholecystectomy is considered the most definitive and reliable. It is endorsed by SAGES, Tokyo Guidelines 2018, and the COSIC framework as the preferred bailout when the CVS cannot be obtained [4,62,78]. Subtotal cholecystectomy is particularly useful when the hepatocystic triangle is obliterated by fibrosis or inflammation, where total cholecystectomy risks bile duct or vascular injury. The principle is to excise as much of the gallbladder as safely possible, ensuring removal of stones, ablation of mucosa in the remnant, and minimization of stump size [79].

Evidence strongly supports subtotal cholecystectomy over attempted total cholecystectomy in hostile anatomy. A meta-analysis of difficult gallbladders found subtotal cholecystectomy significantly reduced bile duct and vascular injuries compared with total cholecystectomy, though at the expense of higher bile leak rates, need for ERCP, intra-abdominal collections, and reoperatio [79,80]. A retrospective analysis of severe cholecystitis cases demonstrated that 10.5% of laparoscopic cholecystectomies required conversion, whereas none of the laparoscopic SCs did. Importantly, bile duct injuries were confined to the total cholecystectomy group. While laparoscopic SC had higher bile leak (13% vs. 3.8%) and subphrenic collection rates (21.7% vs. 6.7%), overall complication and mortality rates were similar [81,82].

Several technical approaches to subtotal cholecystectomy exist. Henneman et al. identified four laparoscopic methods: two excising most of the anterior wall while leaving the posterior wall in situ, and two removing portions of both anterior and posterior walls with transection at Hartmann’s pouch [79]. The gallbladder remnant may be closed (reconstituting subtotal cholecystectomy) or left open (fenestrating subtotal cholecystectomy). To simplify terminology, Strasberg et al. proposed these two main categories [78]. Fenestrating subtotal cholecystectomy reduces the risk of symptomatic remnants and recurrent stones but is associated with higher bile leak rates. Reconstituting subtotal cholecystectomy, conversely, lowers bile leak risk but may predispose to recurrent gallstones or “remnant gallbladder” syndrome.

Reported outcomes highlight these trade-offs. In Henneman’s systematic review, bile leaks were more frequent when the cystic duct was left open (16%) compared to when closed (5.6%), and ERCP was required in 16% versus 2.7%, respectively [79]. Long-term follow-up revealed gallstone recurrence in ~5% of reconstituting subtotal cholecystectomies, which remains acceptable given that post-cholecystectomy syndrome after standard laparoscopic cholecystectomy occurs in 10-40%. Elshaer et al., in a review of 1,231 patients, found significantly higher bile leak rates in open stumps (42% vs. 16.5%) but no significant difference in mortality or major complications [81]. A more recent cohort comparing fenestrating and reconstituting subtotal cholecystectomy showed similar rates of bile duct injury, bile leak, and readmission, but choledocholithiasis was significantly higher in reconstituting subtotal cholecystectomy (6.7% vs. 0.9%) [83].

Comparisons with open cholecystectomy reinforce the advantages of laparoscopic subtotal cholecystectomy. Ramirez-Giraldo et al. found subtotal cholecystectomy had lower bile duct injury, bleeding, intestinal injury, and wound infection rates compared with open cholecystectomy, though bile leaks were more frequent [84]. A 2024 meta-analysis by Aloraini et al. confirmed laparoscopic subtotal cholecystectomy had shorter operative times, shorter hospital stays, and fewer wound infections compared with open cholecystectomy, with similar rates of bile leak and need for postoperative interventions [85]. Kaplan et al. reported zero bile duct injuries in the laparoscopic subtotal cholecystectomy group compared with 3.3% in open cholecystectomy, with fewer severe complications overall [86].

Overall, both fenestrating and reconstituting subtotal cholecystectomies are safe alternatives to total cholecystectomy in difficult cases, each with distinct postoperative risks. Despite higher bile leak rates, fenestrating subtotal cholecystectomy reduces long-term recurrence, while reconstituting subtotal cholecystectomy carries the opposite trade-off. With bile leaks usually manageable by ERCP or drainage, and remnant gallbladder stones often treated endoscopically, subtotal cholecystectomy remains the most definitive and widely accepted bailout strategy. Both Tokyo Guidelines 2018 and IRCAD (*Institut de Recherche contre les Cancers de l'Appareil Digestif*/Research Institute against Digestive Cancer) consensus statements strongly endorse its role. Clear operative documentation is essential, and surgeons should be well-versed in technical nuances and follow-up care [62,87].

Fundus-First (Dome-Down, Retrograde) Approach

The fundus-first or dome-down approach is another valuable bailout technique. Here, dissection begins at the gallbladder fundus and proceeds retrogradely toward the cystic duct, bypassing the hazardous hepatocystic triangle. This technique has been shown to reduce conversion rates and bile duct injury in difficult cases. A Swedish multicenter series involving 1,745 laparoscopic cholecystectomies reported bile duct injury in only 0.07% with the fundus-first versus 0.9% with the conventional approach, with shorter operating times and fewer complications overall [88]. The meta-analyses by El-Boghdady confirm that fundus-first dissection is associated with lower conversion and bile duct injury rates compared with antegrade dissection [89]. Nonetheless, the technique is not without risks. In scleroatrophic gallbladders, fibrosis obliterates the cystic plate, bringing the right portal pedicle dangerously close, the so-called “pucker sign”. In such cases, straying from the gallbladder wall can result in catastrophic vascular injury [19]. Thus, fundus-first should be applied only by surgeons with expertise in hepatocystic anatomy and with readiness to convert to subtotal cholecystectomy if safe progression cannot be maintained.

Taken together, these bailout strategies emphasize judgment over persistence. Whether by aborting, converting, draining, performing subtotal, or adopting a fundus-first approach, the guiding principle remains the same: avoiding bile duct or vascular injury is paramount. Among these, subtotal cholecystectomy is the most definitive and reliable option, but each has a role depending on patient condition and intraoperative findings.

Limitations 

This review has certain limitations that must be acknowledged. Although an effort was made to include the most relevant and up-to-date evidence, it was not possible to review all available literature on the subject. The focus was primarily on difficult laparoscopic cholecystectomy in the context of acute and chronic cholecystitis, where operative difficulty and risk of bile duct injury are most commonly encountered. However, several other clinical scenarios associated with difficult laparoscopic cholecystectomy were not explored in detail. These include procedures performed during pregnancy, in patients with morbid obesity, in those with a history of previous upper abdominal surgery, in the setting of liver cirrhosis or portal hypertension, and in cases of ectopic or abnormally positioned gallbladders. Each of these conditions carries unique anatomical and physiological challenges that warrant specific consideration, but they fall outside the scope of this review.

## Conclusions

Biliary injury after laparoscopic cholecystectomy remains a serious complication with lasting morbidity. To reduce this risk, surgeons must adopt the COSIC, a framework that emphasizes careful anatomical understanding, obtaining the critical view of safety, judicious use of bailout strategies, intraoperative imaging, and timely time-outs. Seeking a second opinion in difficult cases, using energy devices safely, and documenting operative details are equally important pillars of safe practice.

A key principle is that a subtotal cholecystectomy, though incomplete, is always safer than a hazardous “over-dissection” that includes part of the bile duct. Similarly, a controlled bile leak from dissection close to the gallbladder wall is far preferable to an inadvertent bile duct injury. By anticipating operative difficulty, adhering to the ABCD of safe laparoscopic cholecystectomy, and practicing COSIC routinely, surgeons can lower the rate of biliary and vascular injury to levels seen before the laparoscopic era, if not eliminate them entirely.
